# Commentary on Harding *et al*.: Responding to ketamine use disorder—Integrating practice, research, and whole‐system approaches to harm reduction

**DOI:** 10.1111/add.70181

**Published:** 2025-08-27

**Authors:** Rob Ralphs, Janine Day, Jonathan Dewhurst

**Affiliations:** ^1^ Manchester Metropolitan University ‐ School of Sociology and Criminology Manchester UK; ^2^ Early Break, Young People and Family Substance Use Service Bury UK; ^3^ Greater Manchester Mental Health NHS Foundation Trust Manchester UK

**Keywords:** harm reduction, ketamine, mental health, self‐medication, treatment, whole‐system approach

*A whole‐system approach is needed to tackle ketamine use disorder, focusing on public education, early detection of harm and tailored treatment. Current barriers include low awareness, physical symptoms and lack of specialised care. Integrated, person‐centred services and further research into mental health links and treatment strategies are required*.

Harding and colleagues [1] recent study contributes to an established body of international evidence [2, 3, 4, 5, 6] on the physical harms associated with ketamine use disorder (KUD), including bladder dysfunction, abdominal pain (‘K‐cramps’), nasal complications from insufflation and abstinence symptoms such as cravings, low mood, anxiety and irritability. More significantly, the study offers valuable insights into attitudes toward treatment services, revealing widespread reluctance to engage and dissatisfaction among those who did. These findings underscore the need to improve awareness, accessibility and responsiveness of treatment services for individuals with KUD, ensuring they are better aligned with users' needs and expectations.

### A whole‐system approach to early identification and intervention

A whole‐system response is required to address increases in KUD, encompassing public and professional awareness, early harm identification, timely treatment engagement and the development of ketamine‐specific treatment protocols and harm reduction strategies [6, 7]. At the primary prevention level, education on ketamine‐related risks, particularly urological damage and early warning signs, is essential. The use of ketamine for self‐medication in cases of depression underscores the need for public education on the potential mental health benefits of microdosing, in contrast with the risks associated with higher, recreational doses. Harding *et al*. [1] found that 59% of respondents reported insufficient awareness within educational and peer settings regarding the risks of ketamine use. To reduce harm and improve engagement with treatment services, targeted awareness campaigns are needed. These should inform the public about early signs of harm and available treatment options, while also equipping professionals with the knowledge to respond effectively. Healthcare professionals, often the first point of contact, require training to recognise KUD and respond appropriately. In Harding et al.'s [1] study, two‐thirds of those who sought treatment cited health problems as the primary motivator, with 26% attending Accident and Emergency, 25% consulting a general practitioner (GP) and 12% seeing an urologist. These findings highlight the need for integrated care pathways, including screening tools, referral protocols and mental health assessments [7, 8]. Specific guidance for GPs and frontline staff is important to support early identification and intervention, potentially reducing long‐term physical harm and improving treatment uptake

### Tailored and integrated treatment approaches

Effective treatment for KUD requires integrated and tailored approaches that address underlying mental health conditions rather than focusing solely on substance use. Commissioners and service providers should consider differentiated treatment pathways for specific populations, particularly young people [7] and LGBTQI+ [8]. Given growing concerns about earlier onset and associated harms, tailored interventions for younger users are essential. Harding *et al*. [1] suggest that social media may be an effective platform for engaging children and young adults. However, such approaches must be co‐produced and carefully balanced to ensure credibility and relevance [7, 8, 9].

People with KUD present unique challenges to engagement, including frequent urination and severe abdominal pain (‘K‐cramps’), which can hinder attendance and participation in treatment [7]. These physical symptoms must be considered when designing accessible and responsive services. Tailored interventions that accommodate these barriers, such as flexible appointment scheduling, remote support options and trauma‐informed care, may improve engagement and outcomes. A more inclusive and person‐centred treatment offer is essential to meet the diverse needs of individuals with KUD and to ensure equity in access and effectiveness of treatment.

### Toward a future research agenda

Despite increasing prevalence, treatment admissions and drug seizure data indicating a rise in ketamine use and KUD, the underlying drivers of this trend remain poorly understood in the United Kingdom and globally. Several areas warrant further investigation to expand the evidence base, enhance treatment strategies and improve support for individuals with KUD. Harding *et al*. [1] report that 59% of respondents had a diagnosed mental health disorder, an association that warrants deeper exploration. Rising rates of anxiety and depression [10, 11], alongside growing evidence of ketamine's efficacy in treating conditions such as treatment‐resistant depression and acute suicidal ideation [12, 13, 14, 15, 16, 17], may be contributing factors in self‐medication. However, the relationship between KUD and mental health remains underexplored. Future research should examine whether ketamine is used as self‐medication in response to inadequate access to timely, appropriate mental health care. Additionally, treatment approaches that address both substance use and co‐occurring mental health conditions require evaluation. A recent review of pharmacological treatments for KUD [18] highlights the need for a more robust and diverse evidence base. Current research is overly reliant on case studies [1, 18], with a lack of clinical trials and qualitative studies exploring the experiences of ketamine users and treatment professionals.

## AUTHOR CONTRIBUTIONS

**Rob Ralphs:** Writing—review and editing (lead). **Janine Day:** Writing—review and editing (supporting). **Jonathan Dewhurst:** Writing—review and editing (supporting).

## DECLARATION OF INTERESTS

None.

## Data Availability

Data sharing not applicable to this article as no datasets were generated or analysed during the current study.
